# First-rank symptoms: Past, present and future

**DOI:** 10.1192/j.eurpsy.2021.1455

**Published:** 2021-08-13

**Authors:** B. Jorge, C. Pedro Fernandes, M. Duarte Mangas, J. Carvalho

**Affiliations:** 1 Serviço De Psiquiatria, Hospital de Braga, Braga, Portugal; 2 Psychiatry, Hospital de Braga, Braga, Portugal

**Keywords:** schizophrénia, first rank symptoms

## Abstract

**Introduction:**

Conceptualising Schneider’s first-rank symptoms (FRS) as a diagnostic test whose performance can be measured in terms of sensitivity and specificity involves some issues that require reflection. The first formal proposal was contained in a 1939 monograph Schneider wrote, but little is known of their prehistory. In recent years there has been renewed interest in their clinical value.

**Objectives:**

This work aims to review the the diagnostic the evolution and diagnostic accuracy of FRS.

**Methods:**

A non-systematic review was performed, searching Pubmed/MEDLINE for articles using the keywords “schizophrenia” and “first rank symptoms”.

**Results:**

From the beginning of Western descriptive psychopathology in the early 19th century, symptoms have been observed later described as first-rank by Schneider. When FRS are conceived as simple clinical indicators at a low level of inference, the results of the meta-analytic estimate of their diagnostic accuracy can be considered as a valid appraisal of their performance and usefulness. However, when FRS are conceptualised from a psychopathological perspective as strange and incomprehensible experiences that cannot be reduced merely to their propositional content and require substantial expertise and skill to be properly evaluated, the meta-analytic estimates can hardly be seen as a valid evaluation of their diagnostic significance, considering that some FRS are extremely difficult to assess properly.

**Conclusions:**

The descriptions of these symptoms present substantial temporal and geographical continuity, over two centuries and in many countries. There is contradictory information concerning the validity of FRS as a clinical indicator. Phenomenologically informed studies are needed to address this research gap.

